# Myocardial active strain energy density and contractance: novel prognostic tools for left ventricular function and cardiovascular risk

**DOI:** 10.1093/ehjimp/qyaf105

**Published:** 2025-10-06

**Authors:** David H MacIver, Henggui Zhang, David Oxborough, Steffen E Petersen, Nay Aung

**Affiliations:** Biological Physics Group, Department of Astronomy and Physics, University of Manchester, Manchester M13 9PL, UK; Department of Cardiology, Taunton and Somerset Hospital, Taunton TA1 5D, UK; Biological Physics Group, Department of Astronomy and Physics, University of Manchester, Manchester M13 9PL, UK; Research Institute for Sports and Exercise Science, Liverpool John Moores University, Liverpool WA8 3BN, UK; William Harvey Research Institute, NIHR Barts Biomedical Research Centre, Queen Mary University London, Charterhouse Square, London EC1M 6BQ, UK; Barts Heart Centre, St Bartholomew’s Hospital, Barts Health NHS Trust, West Smithfield, London EC1A 7BE, UK; William Harvey Research Institute, NIHR Barts Biomedical Research Centre, Queen Mary University London, Charterhouse Square, London EC1M 6BQ, UK; Barts Heart Centre, St Bartholomew’s Hospital, Barts Health NHS Trust, West Smithfield, London EC1A 7BE, UK

**Keywords:** contractance, contractility, contractile function, energy, heart failure, systolic function, stroke work

## Abstract

Myocardial active strain energy density (MASED), also known as contractance, is a novel measure of myocardial contractile function, defined by the area within the stress–strain loop; it quantifies the energy per unit volume of myocardium used to perform work. MASED applies the principle of strain energy density, which is grounded in engineering science, to cardiac tissue. Using cardiovascular magnetic resonance imaging, we demonstrate that global longitudinal active strain energy density (GLASED), a subtype of MASED, provides superior predictive value compared to conventional metrics such as ejection fraction and global longitudinal strain in predicting mortality among patients with hypertensive heart disease, dilated cardiomyopathy, and amyloid heart disease (*n* = 183). In a large community-based cohort (*n* = 44 957), GLASED was the strongest independent predictor of all-cause mortality and major adverse cardiovascular events among 23 left ventricular structural and functional metrics. Echocardiographic assessment of GLASED further revealed significant associations with age and sex in healthy individuals. These findings indicate that MASED, and specifically GLASED, provide a more accurate and mechanistically grounded assessment of left ventricular performance and cardiovascular risk than established measures. In clinical practice, MASED has the potential to enhance risk stratification, guide heart failure management, and differentiate pathological from physiological hypertrophy. Prospective prognostic studies in wider disease populations are warranted to validate its clinical utility.

## Graphical Abstract

**Graphical Abstract:**
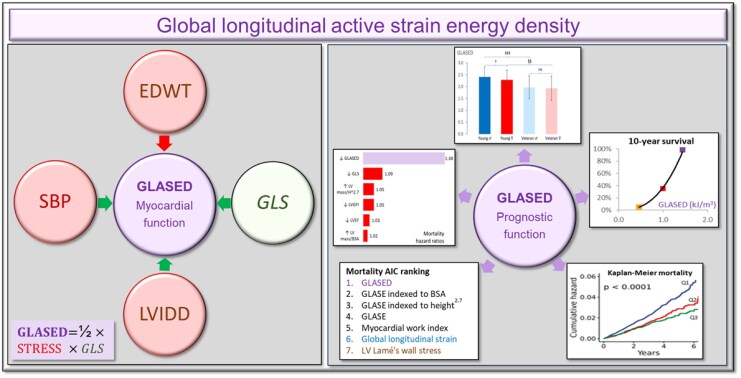
Left panel: GLASED measures myocardial function and is calculated from the longitudinal stress and GLS. Longitudinal stress is determined by the geometric factor and SBP. The geometric factor combines the end-diastolic wall thickness (EDWT) and left ventricular internal end-diastolic diameter (LVIDD). Right panel: GLASED has a prognostic function predicting mortality better than all other known metrics and shows age and sex differences.

## Introduction

An accurate method for evaluating left ventricular (LV) performance is essential for enhancing our understanding of pathophysiological mechanisms, assessing prognosis, and improving management strategies in cardiovascular conditions, particularly heart failure. Therefore, there is a pressing need for more precise measures of cardiac function and outcome prediction.

While more established measures such as LV ejection fraction (LVEF),^1^ myocardial strains,^2,3^ myocardial work index (MWI),^4^ pressure–strain product (an estimate of MWI),^5^ LV global function index,^6^ and myocardial contraction fraction^7^ are available, these tools have inherent limitations in terms of engineering physics as described in more detail below.

Many metrics have attempted to assess prognosis or measure LV myocardial function. Although not the primary focus of this review, a summary of some of the key prognostic studies with references are shown in the Supplement (see Supplementary data online, *Table S1*). Most of these studies have assessed the relative importance of a single metric in risk assessment and many have produced inconsistent results. For example, the LVEF has been shown to be a poor predictor of risk in heart failure.^1,8,9^ Similarly, global longitudinal strain (GLS) is predictive particularly when added to clinical variables or a wall motion score.^10^ LV concentric hypertrophy is also an important risk factor.^11^

There is an increasing drive to improve the assessment of LV function^2^ while avoiding the impact of confounding factors such as load dependence and geometric changes. The development of LV metrics that integrate established prognostic determinants (myocardial deformation, LV enlargement, and concentric remodelling) holds particular promise for enhancing risk stratification. Such composite measures may better capture the multi-factorial pathophysiology underlying adverse cardiac outcomes compared with traditional unidimensional empirical parameters. Until recently, there have been very few studies that directly compare the efficacy of multiple different LV structural or functional metrics on outcome.

This review aims to critically evaluate the theoretical foundations, methodological considerations, supporting data, and emerging clinical evidence for myocardial active strain energy density (MASED) as a group of novel metrics for assessing LV function and cardiovascular risk.

## Engineering background of strain energy density

Energy represents the ability to perform work and drive motion. Strain energy density, a key concept in physics, measures the stored energy per unit volume during compression or stretching of any material and is calculated from the area within stress–strain relationship.^12^ Stress assesses the force per unit area and strain measures the change in shape or deformation. During deformation of an elastomer, chemical bonds are distorted from their equilibrium, storing energy. Upon removing the external force, this energy is released as the material returns to its original shape, enabling elastic recovery. These principles apply whether forces are externally applied or internal from the contractile apparatus. Strain energy calculations are derived directly from work, defined by force exerted over distance, and we have provided the algebraic proof in the Supplementary data online. These concepts are important in evaluating myocardial function because the conversion of chemical energy (adenosine triphosphate) drives the kinetic energy of myocardial motion and generates pressure energy, enabling the ejection of stroke volume. Muscle contraction occurs due to changes in the inter-atomic and inter-molecular bonds in the actin–myosin complex. Therefore, quantifying the myocardial energy that is converted to work offers a more complete assessment of LV mechanical performance when compared with other metrics.

## Contractance and myocardial active strain energy density

Contractility remains a challenging concept to define rigorously and has historically been difficult to quantify with numerous different methods used.^12^ Recently, contractance has been proposed as a novel metric of contractile function, designed to address these limitations. This parameter is derived by integrating the area under stress–strain curves (see Supplementary data online, *Figures S1* and *S2* for *ex vivo* and *in vivo*, respectively).^12^ Contractance quantifies the work done (or energy converted) per unit volume of myocardium as chemical energy is transformed into motion by the contractile tissues and we posited that MASED quantifies myocardial function.^12^

**Figure 1:**
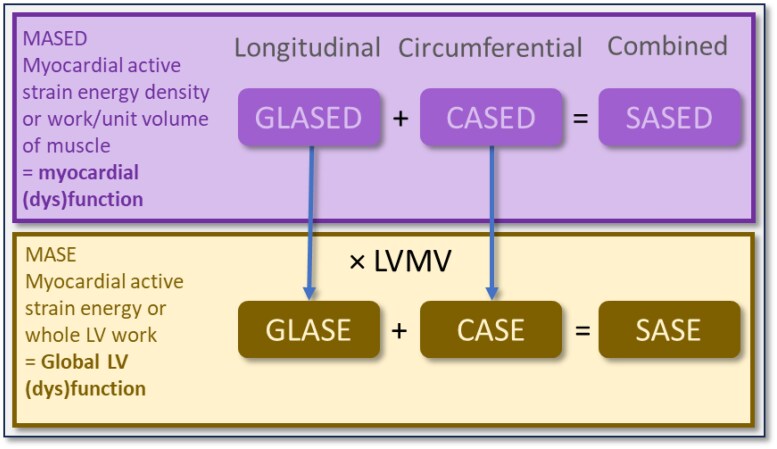
Nomenclature for left ventricular energetics based on myocardial active strain energy and its densities. The figure summarises the nomenclature used in this review. Upper panel: MASED evaluates myocardial contractile (dys)function and can be measured in the longitudinal direction (GLASED) or the circumferential direction (CASED). SASED is the sum of the GLASED and CASED. Lower panel: MASE, obtained by multiplying the MASED by the muscle volume, consequently adjusting for any change in LV muscle mass such that hypertrophy increases MASE (i.e. GLASE, CASE, and SASE). MASE is, therefore, a measure of global left ventricular (dys)function. LVMV, left ventricular muscle volume.

*In vivo*, LV contractance can be measured via MASED. MASED is normally measured in either the longitudinal direction, namely, the global longitudinal active strain energy density (GLASED), or in the circumferential direction, the circumferential active strain energy density (CASED) (*Figure 1*).

**Figure 2:**
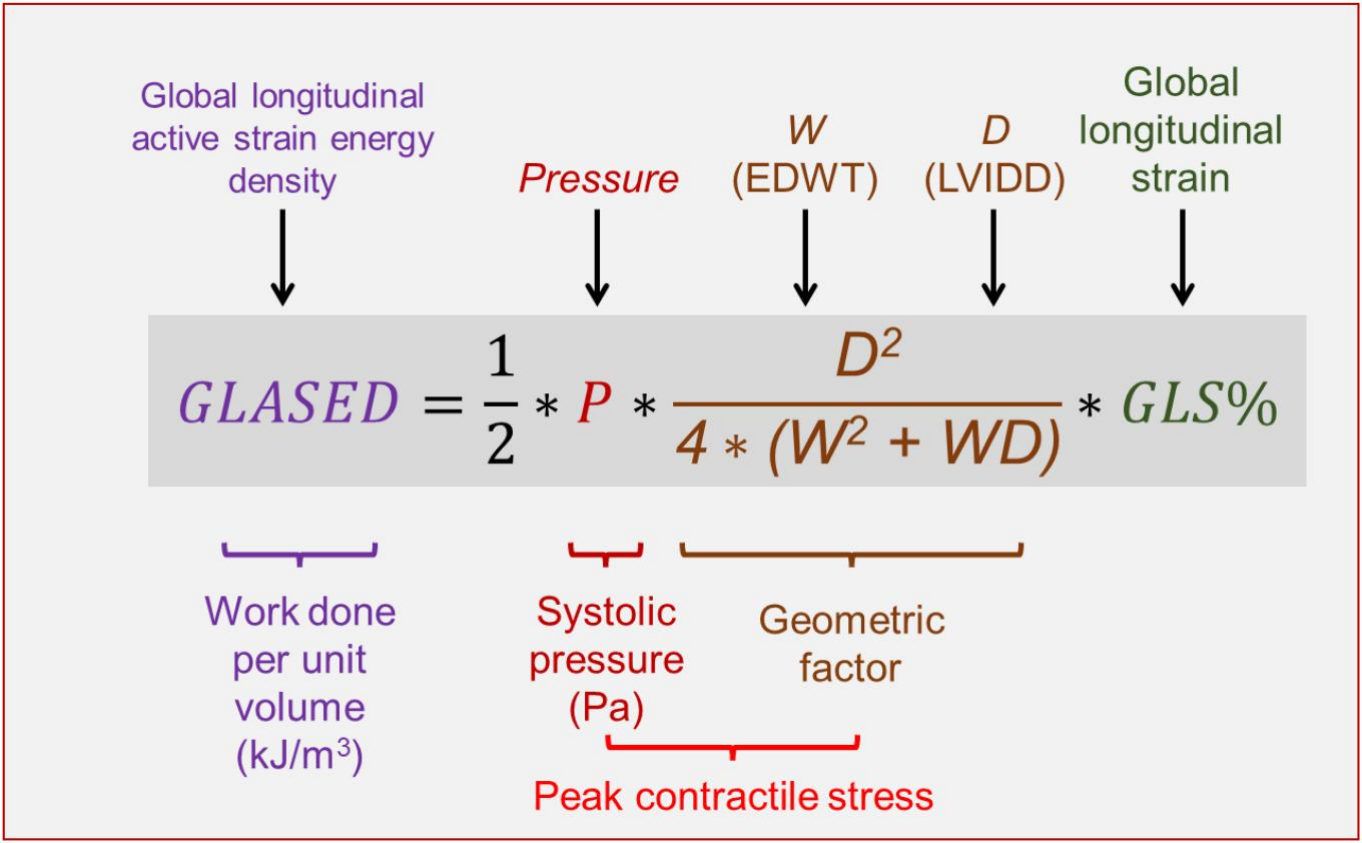
Calculation used for GLASED. GLASED is calculated using the 4 input variables left ventricular end-diastolic diameter (LVIDD), end-diastolic wall thickness (EDWT), systolic blood pressure (*P*), and peak global longitudinal strain (*GLS%*). The equations for GLASED and CASED using the alternative input variables LV inner and outer radii are shown in the Supplement. The longitudinal wall stress (σ_L_) calculations used the Lamé engineering stress and is derived from the geometric factor (brown coloured text) and the systolic blood pressure (as a surrogate for peak systolic LV pressure).

GLASED and CASED quantify the work done per unit volume of myocardium in the longitudinal and circumferential directions respectively by combining information from the relevant myocardial strain and wall stress (*Figure 2*). These quantities are scalar and, therefore, can be added to obtain the sum of the active strain energy densities (SASED) to provide the total myocardial work done per volume of LV myocardium (*Figure 1*).

## Physiological explanations for the limitations of LVEF and strain

The LVEF uses luminal information without reference to LV geometry. The reduced predictive ability of LVEF^2^ is explained by the impact of changes in geometry on LVEF. For example, changes in end-diastolic wall thickness^1,13^ and internal dimensions affect LVEF independent of myocardial strain.^14,15^ Firstly, increasing strain causes an increase in LVEF in a curvilinear manner.^13,14^ The effects of mid-wall-circumferential shortening has approximately twice the impact of long-axis shortening on LVEF and stroke volume.^16^ Secondly, increasing wall thickness increases absolute wall thickening, which in turn independently increases LVEF^13^; and finally, a greater end-diastolic diameter mitigates the influence of absolute wall thickening on LVEF, thus resulting in a lower LVEF.^17^ Our original findings on the consequences of geometric alterations on LVEF have been confirmed by other groups.^18,19^ The effect of geometry on LVEF can be removed by using the corrected ejection fraction (EF_c_); however, we found the EF_c_ provides no additional prognostic benefit over strains.^20^

Experimental studies using *ex vivo* cardiac trabeculae have demonstrated key limitations of relying solely on strain measurements.^12^ Inotropic stimulation of healthy myocardium increases contractile stress, strain, and MASED (see Supplementary data online, *Figure S3A*). However, when stress demand increases, peak strain decreases because the myocardium cannot generate sufficient contractile force (stress capacity) to achieve normal deformation (see Supplementary data online, *Figure S3A*). Conversely, reducing afterload (stress demand) improves strain by lowering the required contractile effort.^12^ Moreover, these experiments revealed an optimal stress and strain operating range for maximal work (contractance) and myocardial performance (see Supplementary data online, *Figure S3B*).

Further, it should be highlighted that the afterload effects on strain secondarily alters LVEF as described above. The changes in strain with altered afterload are accompanied by changes in contractile stress and vice versa emphasizing the importance of combining stress and strain information with MASED and avoiding using either strain or stress data in isolation.

## Prognostic studies: mortality and major adverse cardiovascular events

**Figure 3:**
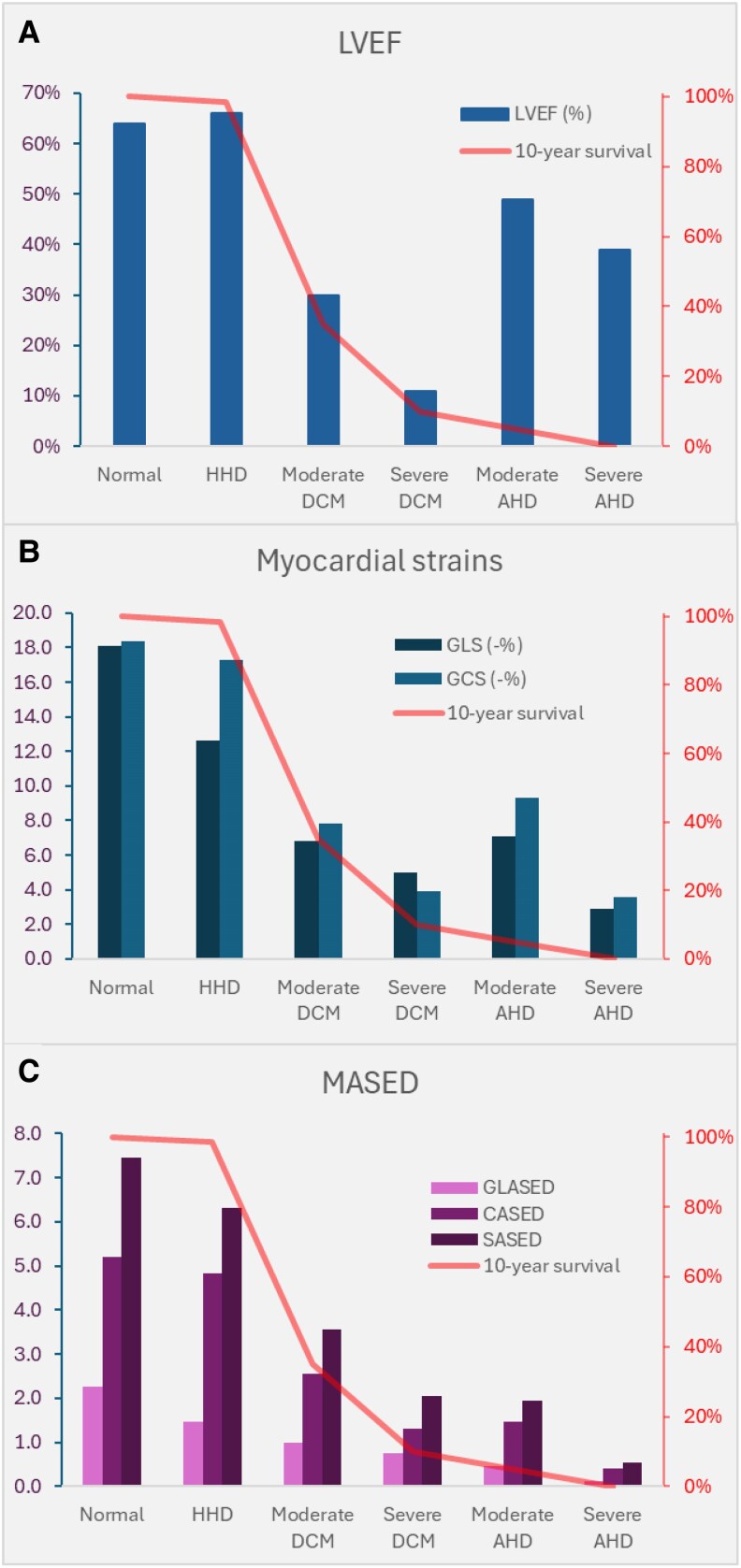
LVEF (*A*), strains (*B*), GLASED, CASED, and SASED (*C*) compared with expected 10-year survival in different disease cohorts. (*A*) There is a poor relationship between LVEF and expected survival especially in thicker-walled disorders (HHD and AHD). (*B*) GLS and GCS perform particularly weakly in AHD. (*C*) GLASED, CASED, and SASED achieve better expected survival prediction in all the cohorts. HHD, hypertensive heart disease; DCM, dilated cardiomyopathy; AHD, amyloid heart disease. Expected survival was based on estimates from similar disease cohorts. GLASED is based on Lamé equations for stress using longitudinal strain rather than long-axis shortening. These data have been published previously.^20,30^

Given a likely link between LV myocardial function and assessing prognosis, we undertook an exploratory study (*n* = 183), assessing GLASED, CASED, and SASED in cohorts with hypertensive heart disease (HHD), dilated cardiomyopathy (DCM), and amyloid heart disease (AHD) via CMR.^20^ The results were compared with the expected survival rates in these disease cohorts. GLASED, CASED, and SASED outperformed all other metrics. LVEF markedly underestimated the risk in AHD (*Figure 3*). GLS and global circumferential strain (GCS) outperformed LVEF but still moderately underestimated the risk in AHD.^20^ The MASED subtypes, because they account for the contractile stresses, outperforms strain.^12^ GLASED and CASED also predicted mortality better than the corrected LVEF,^21^ longitudinal and circumferential stresses, longitudinal and total internal forces, stroke work and indexed stroke work,^22^ and pressure–strain product (MWI)^23^ in predicting expected outcomes.^20^ The lower prognostic value of the pressure–strain product compared with that of MASED likely reflects the limitations of using luminal pressure compared with a contractile stress (see below for more details).

In a subsequent study, 23 possible prognostic metrics were assessed retrospectively via cardiovascular magnetic resonance imaging (CMR) in a large community-based cohort of 44 957 individuals analysed using a fully convoluted neural network.^24^ To our knowledge, this is the largest and most comprehensive comparative study to date that assesses multiple potential prognostic metrics. The ideal prognostic metric should demonstrate: (i) Strong model discriminability with superior Akaike information criterion (AIC) ranking, (ii) Robust prognostic power with significantly elevated hazard ratio (HR), and (iii) Clinically meaningful stratification (Kaplan–Meier survival curves with distinct separation and highly significant log-rank *P*-values).

**Figure 4:**
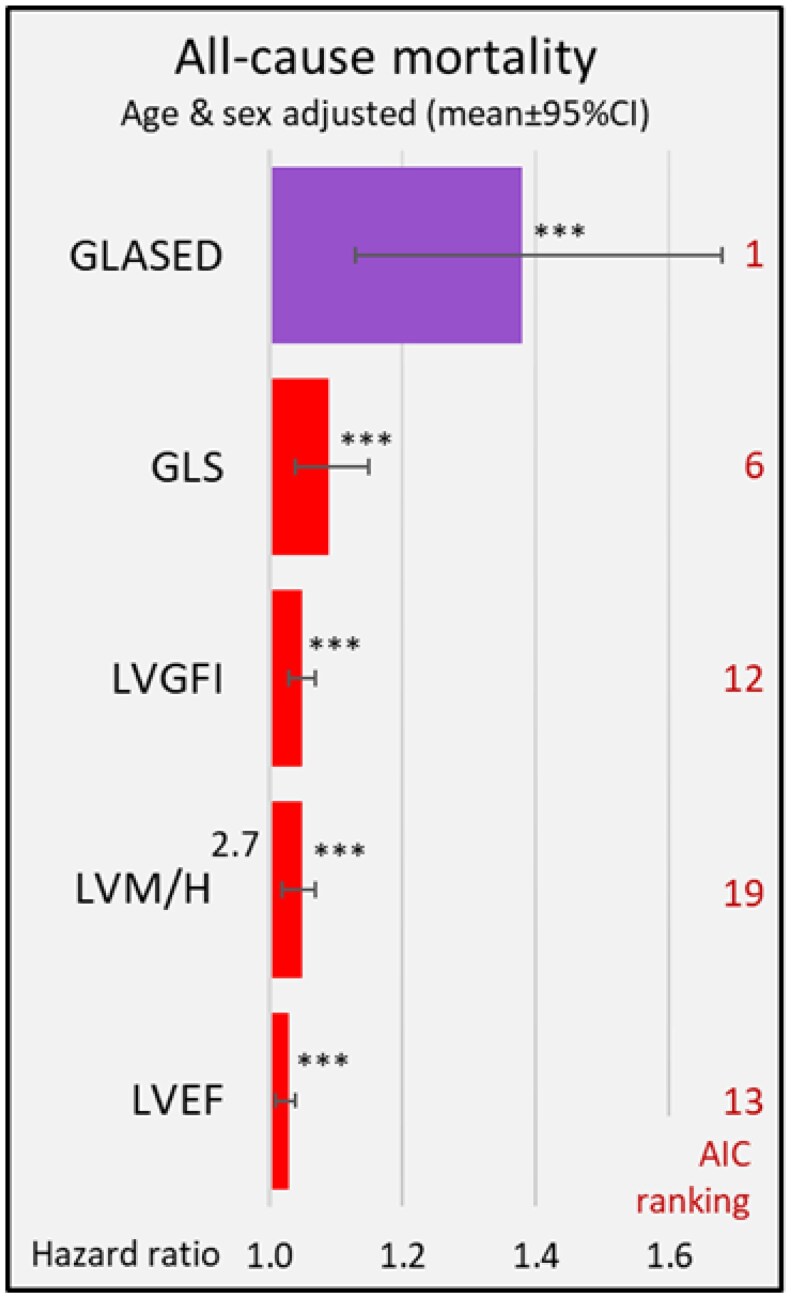
Age- and sex-adjusted all-cause mortality HRs and rankings for GLASED and other potential prognostic metrics in a low-risk community-based cohort. GLASED had the highest HR (1.38) and performed better than all other metrics. Furthermore, GLASED was ranked first by the AIC; stroke work and pressure–strain product (MWI) had HRs of 1.0 (n.s.)^24^. ***Indicates *P* < 0.0001.

**Figure 5:**
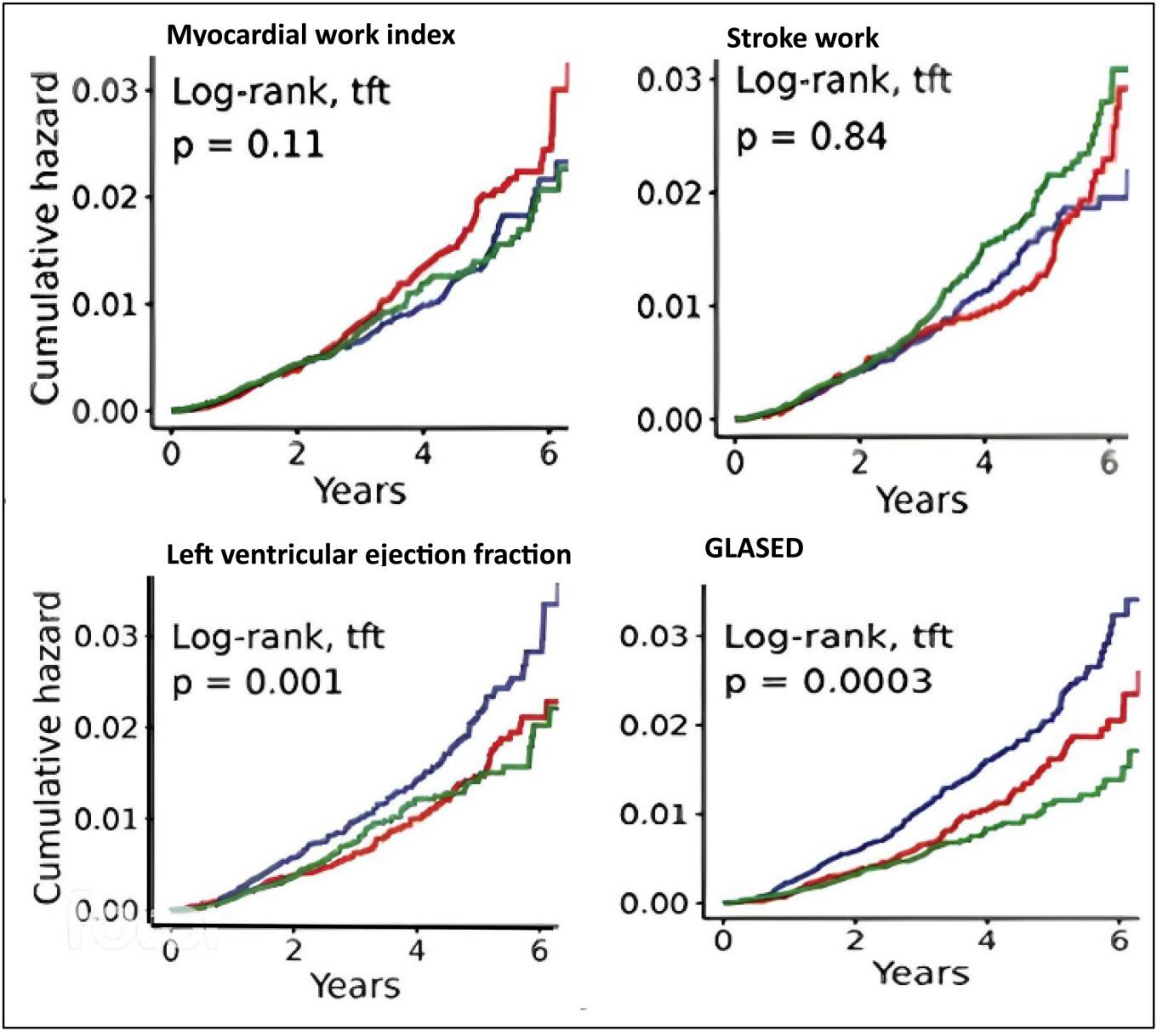
Kaplan–Meier all-cause mortality curves for pressure–strain product, stroke work, LVEF, and GLASED in low-risk community-based cohort.^13^ The figure shows the MWI (derived from the pressure–strain product) and stroke work did not predict time to first event. LVEF did not differentiate mortality between the lower two tertiles. GLASED accurately predicted risk with separation of the three tertiles and with the highest level of significance of all 23 metrics.

GLASED demonstrated the highest HR for all-cause mortality (*Figure 4* and *Table 1*) and major adverse cardiovascular events (MACE) compared with GLS, LVEF, and the 20 other potential structural or functional metrics (*Table 2*). The AIC ranking placed GLASED first out of the 23 potential metrics for both all-cause mortality and MACE (*Tables 1* and *2*). *Tables 1* and *2* also display the Kaplan–Meier curve *P*-values and the number of separate tertile curves adjusted for age and sex. The Kaplan–Meier curves for all-cause mortality (*Figure 5*) did not show separation for MWI or stroke work but there was good separation across all three tertiles of GLASED (*Figure 5* and *Table 1*). Comparable results were obtained whether the analyses were adjusted for age and sex alone, or for age, sex, and all cardiovascular risk factors.^24^ These positive for results for GLASED are particularly surprising given the low risk of the community-based cohort investigated.

**Table 1:** All-cause mortality for each metric (adjusted for age and sex)

Marker (sorted by AIC ranking)	HR	K–M *P*-value	K–M separation	AIC rank
↓ GLASED	1.38*	0.0003*	3*	1
↓ GLASE/BSA	1.00	0.09	1	2
↓ GLASE/height^2.7^	1.00	0.04*	2*	3
↓ GLASE	1.00	0.095	1	4
↓ Pressure–strain product	1.00	0.11	1	5
↓ Global longitudinal strain	1.09*	0.015*	3*	6
↓ LV Lamé wall stress	1.00	0.014*	3*	7
↓ Stroke work/left ventricular mass	1.01*	0.001*	2*	8
↓ Stroke work/height^2.7^	1.00	0.26	1	9
↓ Stroke work/BSA	1.00	0.4	1	10
↓ Stroke work	1.00	0.84	1	11
↓ LV global function index	1.05*	0.0026*	3*	12
↓ LV ejection fraction	1.03*	0.001*	2*	13
↓ LV contraction fraction (SV/LVMV)	1.01*	0.001*	3*	14
↑ LV mass/BSA	1.02*	0.2	1	15
↑ LV mass/height^2.7^	1.05*	0.2	1	16
↑ LV end-diastolic volume/height^2.7^	1.01	0.63	1	16
↑ LV mass	1.01*	0.011*	3*	17
↑ LV end-diastolic volume/BSA	1.00	0.17	1	18
↑ LV end-diastolic volume	1.00	0.73	1	20
↓ LV end-diastolic diameter/BSA	1.00	0.27	1	21
↑ LV end-diastolic diameter	1.02	0.07*	1	22
↑ LV end-diastolic diameter/height^2.7^	1.02	0.37	1	23

HR, hazard ratio; K–M, Kaplan–Meier *P*-value and separation of tertiles. Akaike information criterion (AIC). *Indicates significant Bonferroni adjusted *P*. See Aung^11^ for confidence intervals, ΔAIC values and adjustment for age, sex, and all cardiovascular risk factors.

**Table 2:** Major adverse cardiovascular events (adjusted for age and sex)

Marker (sorted by AIC ranking)	HR	K–M *P*-value	K–M separation	AIC rank
↓ GLASED	1.39*	0.0001*	3*	1
↓ GLASE/H^2.7^	1.01*	0.92	1	2
↓ GLASE indexed to BSA	1.00	0.91	1	3
↓ GLASE	1.00	0.0023*	2*	4
↓ Pressure–strain product	1.00	0.98	1	5
↓ Global longitudinal strain	1.12*	0.0001*	3*	6
↓ LV Lamé wall stress	1.00*	0.0001*	2*	7
↓ Stroke work/LV mass	1.01*	0.0001*	2*	8
↓ Stroke work/H^2.7^	1.00*	0.081	2	9
↓ Stroke work/BSA	1.00	0.81	2	10
↓ Stroke work	1.00*	0.02*	2*	11
↑ LV mass/BSA	1.05*	0.0001*	3*	12
↑ LV mass/H^2.7^	1.10*	0.0002*	3*	13
↑ LV mass	1.02*	0.0001*	3*	14
↓ LV contraction fraction (SV/LVMV)	1.02*	0.0001*	3*	15
↑ LV end-diastolic diameter	1.03*	0.41	2	16
↓ LV end-diastolic diameter/BSA	1.00*	0.0041*	3*	17
↓ LV global function index	1.07*	0.0001*	3*	18
↑ LV end-diastolic diameter/H^2.7^	1.11*	0.012*	2*	19
↓ LV ejection fraction	1.03*	0.0001*	2*	20
↑ LV end-diastolic volume/H^2.7^	1.03*	0.11	1	21
↑ LV end-diastolic volume/BSA	1.01*	0.51	1	22
↑ LV end-diastolic volume	1.00*	0.0073*	3*	23

HR, hazard ratio; K–M, Kaplan–Meier *P*-value and separation of tertiles. Akaike information criterion (AIC). *Indicates significant Bonferroni adjusted *P*. See Aung^11^ for confidence intervals, ΔAIC values and adjustment for age, sex, and all cardiovascular risk factors.

While MASED subtypes show strong prognostic promise, methodological limitations such as retrospective study designs, and limited population diversity should be acknowledged. These factors may affect generalizability and clinical interpretation. Nonetheless, MASED’s integration of hemodynamic load, geometry, and contractile function presents a mechanistically robust alternative to traditional metrics, supporting its potential clinical utility pending further prospective validation (see Supplementary data online, *Figure S4*).

## MASED unites key risk factors

Four of the more important risk factors in LV diseases are a high blood pressure (higher peripheral vascular resistance ± increased LV output), LV mass (wall thickness), LV chamber size/dimensions, and myocardial strains (see Supplementary data online, *Table S1* for details). GLASED synthesizes these independent prognostic variables, i.e. LV internal diameter, SBP, wall thickness, and GLS. Notably, each MASED input variable is a risk marker in its own right. A high blood pressure without remodelling would be expected to increase the wall stress but, in HHD, concentric remodelling occurs resulting in an overall lowering of stress and, for a given strain, decreasing MASED (see Supplementary data online, *Figure S4*).

In DCM, ventricular dilation occurs as a compensatory mechanism to maintain stroke volume despite reduced ejection fraction.^25^ By increasing end-diastolic volume, stroke volume improves for a given LVEF^26^; a process driven by remodelling rather than the Frank–Starling mechanism, which is diminished in heart failure.^25^ We hypothesise that the dilation elevates systolic wall stress requirement (via Laplace’s law) while intrinsic contractile function declines. This results in a mismatch between elevated contractile demand (increased wall stress) and diminished contractile capacity (reduced myocardial force generation ability). Crucially, because of this contractile stress demand-capacity mismatch, the strain falls markedly despite any increased catecholamine drive. The substantial reduction in strain outweighs the small rise in wall stress, resulting in a lower MASED.

This observation provides a premise as to how a dilated ventricle is associated with increased risk; specifically the higher stress demand is accompanied by a lower strain and a reduced capacity to do work. Thus, by capturing the interplay between loading conditions, structural adaptation, and actual myocardial performance, MASED may offer a more integrated and functionally relevant risk metric than any single component.

## Implications for heart failure

GLASED may aid in distinguishing heart failure due to LV myocardial disease from other causes. *Table 3* presents the heart failure risk associated with GLASED compared with other potential risk metrics. GLASE/H^2.7^, GLASE/BSA, and GLASE had AIC rankings of 1, 2, and 3, respectively, for heart failure risk; however, each had a low HR that was not statistically significant, with Kaplan–Meier curves showing no separation across three tertiles. GLASED was ranked fourth, had a statistically significant HR of 1.4, and its Kaplan–Meier curves separated all three tertiles for incident heart failure.

**Table 3:** Heart failure risk (adjusted for age and sex)

Marker (sorted by AIC ranking)	HR	K–M *P*-value	K–M separation	AIC rank
↓ GLASE/height^2.7^	1.02*	0.63	1	1
↓ GLASE/BSA	1.01*	0.45	1	2
↓ GLASE	1.00*	0.27	1	3
↓ GLASED	1.41*	0.035*	3*	4
↓ Global longitudinal strain	1.30*	0.0001*	2*	5
↓ Pressure–strain product	1.00*	0.0075*	2*	6
↓ LV Lamé's wall stress	1.00	0.7	1	7
↓ LV ejection fraction	1.11*	0.0001*	2*	8
↓ LV global function index	1.19*	0.0001*	3*	9
↑ LV end-diastolic diameter	1.16*	0.072	1	10
↓ Stroke work/left ventricular mass	1.02*	0.0001*	2*	11
↑ LV end-diastolic volume/BSA	1.03*	0.6	1	12
↑ LV end-diastolic volume	1.02*	0.46	1	13
↑ LV mass/BSA	1.06*	0.0038*	3*	14
↑ LV end-diastolic volume/height^2.7^	1.08*	0.041*	3*	15
↑ LV mass/height^2.7^	1.14*	0.043*	3*	16
↑ LV mass	1.03*	0.047*	3*	17
↓ LV end-diastolic diameter/BSA	1.15*	0.36	1	18
↓ Stroke work	1.00	0.17	1	19
↓ Stroke work/BSA	1.00	0.16	1	20
↓ Stroke work/height^2.7^	1.00	0.31	1	21
↓ LV contraction fraction (SV/LVMV)	1.04*	0.0001*	2*	22
↑ LV end-diastolic diameter/height^2.7^	1.45*	0.62	1	23

HR, hazard ratio; K–M, Kaplan–Meier *P*-value and separation of tertiles. Akaike information criterion (AIC). *Indicates significant Bonferroni adjusted *P*. See Aung^11^ for confidence intervals, ΔAIC values and adjustment for age, sex, and all cardiovascular risk factors.

Only the LV end-diastolic diameter/H^2.7^ ratio had a higher HR but was AIC ranked last (23rd), and the Kaplan–Meier *P*-value was not significant. GLS performed less well regarding heart failure risk prediction than GLASED with a HR of 1.3, ranking fifth and did not distinguish between all three Kaplan–Meier tertiles.

GLASED is inversely related to the expected BNP.^20^ The expected BNP levels matched GLASED levels in each of the cohorts with myocardial diseases, suggesting that GLASED may also be a good measure of heart failure severity.^20^

## Understanding ventricular work vs. myocardial work

Understanding the distinction between LV work and work indexed to myocardial volume is essential for fully comprehending cardiac performance in various physiological and pathological states. LV work and work indexed to myocardial volume can be assessed using MASE and MASED, respectively. Myocardial active strain energy (MASE) is the energy expended by the LV muscle during contraction and is calculated by multiplying MASED by the LV muscle volume. MASE may be valuable in assessing the overall work of the LV in conditions such as HHD with sufficient compensatory hypertrophy and a failing LV due to inadequate compensatory hypertrophy. Furthermore, it can be postulated that MASE will decrease proportionally to the degree of global ventricular dysfunction, with lower MASE values indicating more severe LV dysfunction and likelihood of heart failure.

Healthy myocardium is anticipated to have a normal MASED. We have shown that in DCM and AHD, both MASED and MASE are reduced suggesting related myocardial and LV dysfunction, respectively.^20^ DCM and AHD have unhealthy hypertrophy. In HHD, the combination of reduced GLASED (indicating myocardial dysfunction) and high GLASE (indicating enhanced global LV work) is secondary to the combination of diseased myocardium and compensatory hypertrophy. The increased peripheral vascular resistance demands more energy for adequate tissue perfusion.

Athletes exhibit normal GLASED (normal myocardial function) but increased GLASE (greater LV work per beat), which aligns with healthy hypertrophy. These findings suggest that GLASED may distinguish pathological from physiological hypertrophy. This implies that GLASED could play a role in screening for myocardial disease in individuals, including athletes, with a borderline phenotype. Patients with hypertrophic cardiomyopathy have systolic dysfunction even with normal or enhanced LVEF^27^ and thus are expected to have reduced MASED.

The combination of MASE and MASED information provides a comprehensive understanding of myocardial performance, allowing for better assessment of intrinsic myocardial function dependent or independent of overall LV mass. This distinction is central for accurately evaluating cardiac function in various clinical scenarios and for understanding the adaptive or maladaptive responses of the myocardium.^25^

## Stroke work vs. MASE

Stroke work/LV mass, stroke work/H^2.7^, stroke work/BSA, and stroke work were ranked 8–11/23 respectively, each had a HR of 1.0 (n.s) and the Kaplan–Meier mortality curves did not separate. The inferior performance of stroke work compared with MASE and MASED is unexpected and requires explanation. It is evident that stroke work measures true work (unlike MWI) and mathematical derivation of stroke work from first principles is shown in the Supplement (Supplementary data online, equations 9 to 14). However, stroke work measures ventricular function indirectly via luminal information alone (i.e. luminal volumes and pressure); whereas, MASE is derived from myocardial information (strain, contractile stress, and myocardial mass). This observation may explain the improved utility of GLASE over stroke work and GLASED over stroke work indexed to the LV mass.^20,24^

## Myocardial work index vs. MASED

The HR for the MWI was 1.00 (n.s.), ranked 5/23 and the Kaplan–Meier mortality curves were not significant.^24^ MWI is higher in AHD than DCM despite a worse prognosis.^20^ The MWI uses luminal pressure and GLS in its calculation but does not incorporate wall stress^5^; despite using SBP in the calculation of MWI, GLS outperforms the MWI with a HR of 1.09 and Kaplan–Meier curve separation for all three tertiles (*P* = 0.015) and a similar ranking of 6/23.^24^ MWI was also no better than GLS in predicting survival in HHD, DCM, and AHD.^20^

The explanation for the mediocre performance of MWI is as follows. Work is defined by a displacement multiplied by force in the *same* direction; however, luminal pressure exerts a force perpendicular to the direction of the longitudinal displacement. In contrast, MASED employs wall stress (and a force) in the same direction as the strain (and displacement) and therefore provides a more meaningful assessment of work than pressure–strain data (see Supplementary data online for mathematical details). The MWI, therefore, does not directly measure either work or work density as the geometric information and stress calculation are absent; hence its units are mmHg% rather than kJ/m^3^ (see Supplementary data online for a full details). As shown in *Figure 2*, the geometric data (in brown) is essential to calculate both work and work density.

Although MWI is proportional to the true work density when the geometry (or more correctly the geometric factor in *Figure 2*) is the same. The presence of high BP in hypertension normally results in ventricular remodelling with a greater mural thickness and, often a slightly lower ventricular volume and diameter^28^; both factors that reduce stress and result in an overestimate of the work density using MWI. Similarly, MWI overestimates work density in any other thicker-walled LV including infiltrative cardiomyopathies.^20^ In contrast, in DCM, MWI will underestimate work density in a dilated ventricle because of the higher contractile stress incurred. The absence of incorporating geometric data in the MWI explains its relative underperformance as a risk metric.

## Methods for measuring myocardial active strain energy density

MASED can be measured by either cardiovascular magnetic resonance imaging (CMR)^20,24^ or echocardiography.^29^ Determining the area within the stress–strain curve, while the gold standard for MASED, is impractical for clinical use. However, a close approximation to MASED can be made using a suitable equation (*Figure 2*).^20^ The calculations for GLASED, CASED, and SASED are provided in the Supplement (Supplementary data online, equations 1, 3, and 5, respectively). Four pieces of information are necessary for their computation: (i) mean wall thickness, ideally the average of the six basal and six mid-wall segments; (ii) mean end-diastolic of dimension preferably at the three basal and three mid-regions; (iii) precise brachial systolic blood pressure (ideally the mean of three readings close to the time of the procedure where possible); and (iv) the relevant strain, either GLS or GCS (preferably measure three times). For example, an individual with a SBP of 120 mmHg, mean EDWT of 8 mm, LVIDD of 53 mm, and a GLS of −20% would have a GLASED of 2.3 kJ/m^3^ The calculations for MASED and MASE can be automated via an online calculator (e.g. GLASED calculator at https://glased-calculator.s3.eu-west-1.amazonaws.com/index.html) or a pre-written spreadsheet (available on request) resulting in immediate and reliable results. It should be emphasized that the quality of the output is determined by the accuracy of the input variables (see Supplementary data online, *Figure S5*).

## Normal values for MASED and MASE

In an echocardiography study, the young male control group (21 years) had a mean GLASED of 2.31 kJ/m^3^ and a reference range of 1.54–3.08 kJ/m^3^. There was a trend to higher GLASED in the young male athletes (GLASED 2.40 kJ/m^3^, n.s.)^29^ Older athletes (mean 54 years) had a mean GLASED of 1.94 kJ/m^3^. Additional reference ranges across different normal and clinical populations are shown in the Supplementary data online.

A CMR study indicates that a normal mean GLASED value for normal controls was 2.27 kJ/m^3^ (mean age 45 years).^20^ There is an increasing mortality risk when the GLASED value falls below 1.5 kJ/m^3^.^20,30^ Normal CASED values are about twice GLASED (5.27 kJ/m^3^) reflecting the higher (almost double) circumferential stress.^20^

The calculation of MASE is dependent on an accurately calculated myocardial muscle volume which varies amongst methods. In our echocardiographic study, we found the normal male controls had a GLASE of 268 mJ (LV mass 121 g from cube equation) and in a CMR study,^24^ it was 205 mJ (LV mass 86 g). The CMR group were mixed sex and older (21 vs. 65 years), which would not explain the difference in LV mass and GLASE and therefore methodological differences were important. The reference ranges for MASED and MASE differ between CMR and echocardiography due to inherent differences in the way of measuring LV mass, strain, wall thickness, and internal dimensions.

## Impact of age and sex on GLASED

**Figure 6:**
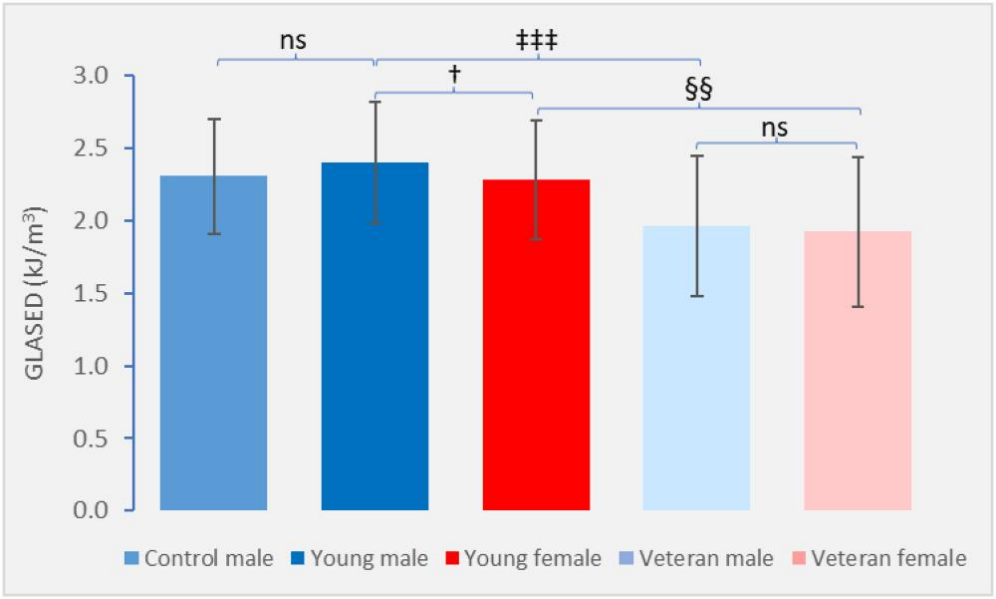
Echocardiographic derived GLASED by age and sex in athletes.^15^ The figure shows that GLASED was higher in young male athletes compared with young female athletes and higher in young athletes compared with veteran athletes.^29^ The GLASED was not statistically significantly different in young control (non-athlete) males compared with young male athletes. GLASED was the same in veteran male and veteran female athletes. ns, not significant; †, *P* < 0.05 for young male athletes vs. young female athletes; §§, *P* < 0.01 Young female athletes vs. Veteran female athletes; ‡‡‡, *P* < 0.0001 for young male athletes vs. veteran male athletes.

Significant age and sex variations have been observed in echocardiographic derived GLASED values (*Figure 6*). In a study with 447 participants, young male athletes demonstrated higher GLASED than young female athletes (2.40 vs. 2.28 kJ/m^3^, mean age of 21.5 years).^29^ No sex difference was observed in veteran athletes (mean age 54 years).^29^ Veteran male had a GLASED of 1.96 kJ/m^3^ and veteran female athletes had the lowest GLASED values (1.92 kJ/m³).^29^

GLASED also decreased with age in the community-based cohort using CMR.^24^ In contrast to the echo study, in an older age group (mean age 64 years), GLASED was higher in females, probably due to the increased prevalence of cardiovascular risk factors and potentially greater subclinical myocardial disease in older males.^24^

## Theoretical advantages and limitations of MASED

*Table 4* summarizes the advantages and limitations of MASED. Various analytic methods can estimate LV wall stress, with Lamé stress showing a strong correlation with stresses derived from finite element modelling and outperforming other methods, including Laplace stress.^30^ Lamé stress provides a more accurate assessment of stress than Laplace because it is more suitable for thick-wall chambers such as the left ventricle.^30^ Employing contractile stress rather than luminal pressure accounts for variations in ventricular geometry, provides a meaningful measurement of the burden on cardiomyocytes, and results in a more accurate calculation of myocardial work than the MWI.^30^

**Table 4:** Theoretical advantages and limitations of contractance and MASED compared with conventional measures

Advantages
Based on established engineering science
Measures work done (energy produced) per unit volume of myocardium
Corrects for the effects of stress on strain
Corrects for the effects of strain on stress
Corrects for mass of tissue e.g. trabeculae vs. papillary muscle
Applicable to and comparable across *in vivo*, *in vitro*, and *ex vivo* studies
Accounts for changes left ventricular geometry including hypertrophy and dilatation
Good predictor of all-cause mortality and MACE
Good predictor of heart failure risk
Considers inotropic state (cf. contractility)
Requires only four clinical measurements
Uses myocardial rather than luminal information (cf. stroke work)
Factors in preload and afterload conditions (cf. contractility)
Integrates known risk factors: LV size and mass, hypertension, and strain
**Limitations**
Lack of familiarity
Requires more clinical measurements to improve accuracy
More time consuming for multiple measurements
Uses sphygmomanometer pressure rather than ventricular pressure
Complexity of calculations
Potential for propagation errors
Limited prognostic information
No data in specific clinical scenarios such as valve and ischaemic disease
Limited data on reference ranges
Reproducibility unknown between a patient, operators, and vendors
Material properties assumed to be isotropic rather than anisotropic
Material properties assumed to be elastic rather than hyperelastic
Additional training required for implementation

Importantly, no correlation exist between stress and strain (*R*^2^ < 0.01) and each provides independent and complementary prognostic information^20,24^ therefore using either stress or strain alone is unreliable. By combining stress and strain information, MASED provides a comprehensive view of myocardial performance, capturing contractile changes that stress or strain alone might miss.^12^ MASED is potentially a more robust measure of myocardial function than contractility because it accounts for Frank–Starling mechanism, systolic pressure and geometric factors as well as the inotropic conditions (see Supplementary data online, *Figures S3A* and *S3B*).^12^ The use of MASED and contractance also improves comparisons between *in vitro*, *ex vivo*, and *in vivo* studies, boosting translational research potential (as demonstrated in Supplementary data online, *Figures S2* and *S4*).

MASED has important limitations, for example unfamiliarity and the need for precise geometric data. The impact of changes in each of the four variables on GLASED is shown in Supplementary data online, *Figures S3*. The more measurements of each variable will lessen the risk of propagation errors. Monte Carlo quantitative error propagation analysis based on single measurements are shown in Supplementary data online, *Table S2*. Performing multiple measurements as outlined above improves GLASED accuracy, reducing the coefficient of variation from 20.9% to 8.8% (see Supplementary data online, *Tables S2* and *S3*). This is similar to the inter-operator coefficient of variation for 2D LVEF (10.3%).^3^ For example, a patient with a GLASED of 2.0 kJ/m^3^, would have a measurement uncertainty of ±0.35 kJ/m³ (1.65–2.35 kJ/m^3^).

MASED may be limited in arrhythmias giving differing values per beat; however, this is also a limitation of most current metrics. Despite these limitations, MASED appears to offer higher prognostic discrimination^20,24^ than traditional metrics (see above).

## Knowledge gaps and future directions

While MASED metrics have considerable potential, several key areas need further investigation to fully realize their application in clinical practice. Standardizing protocols for MASED and MASE (using LV mass) measurements is essential to ensure consistency and reproducibility across various healthcare settings. This includes developing uniform methods across imaging modalities to measure LV muscle volume to enhance MASE accuracy. Better data on relevant reference ranges across ages and sexes are also required.

Further studies are required to assess medication effects and in different clinical scenarios for instance hypertrophic cardiomyopathy, heart failure with preserved ejection fraction, arrhythmias including atrial fibrillation, non-compaction cardiomyopathy, valve, and ischaemic disease (with regional wall motion abnormalities). In addition, cost-effectiveness analysis comparing MASED calculation requirements vs. simpler biomarker approaches would be prudent. At present GLASED is measured using GLS, which includes information from the apex, whereas the dimension data are from the basal and mid-regions; future studies should include assessment of segmental and regional active strain energy densities.

Long-term prospective validation studies are necessary to compare the clinical utility of MASED and MASE with the other metrics as well as wall motion scores, blood biomarkers, myocardial tissue characterization using T1/T2 mapping, and late gadolinium enhancement. A comparison of MASED with myocardial metabolism assessed using nuclear methods would also be of interest.

To enable widespread adoption, strategies must be developed to integrate MASED into routine clinical practice seamlessly. Methodological innovations such as machine learning, automated analysis tools and specialized software will further enhance input variable accuracy and reduce the time necessary for extraction of input variables and automate the calculations (see GLASED calculator https://glased-calculator.s3.eu-west-1.amazonaws.com/index.html) to minimize errors and improve MASED precision.

Long-term longitudinal studies are crucial for assessing the ability of MASED and MASE to predict and monitor cardiovascular outcomes over extended periods. Studies involving multi-variable analyses including established cardiovascular risk factors (NT-proBNP, eGFR, diabetes, prior MI, and medication classes), different demographic/ethnic profiles and to determine if there is any incremental prognostic value over recognized risk scores such as MAGGIC and Seattle Heart Failure scores are to be encouraged.

## Conclusions

Despite the potential methodological limitations of MASED, it may be timely to consider complementing traditional, empirically derived measures of myocardial function such as LVEF, myocardial contraction fraction, MWI, and global function index with metrics that are grounded in engineering science and biophysical principles. Integrating such approaches could provide additional mechanistic insight and potentially enhance the physiological relevance and reproducibility of cardiac functional assessment; this will be particularly important in the research setting.

Measuring strain in the absence of stress information offers an incomplete picture. MASED, by integrating stress and strain, may be a better metric for evaluating myocardial performance and has promising prognostic value in various cardiovascular conditions.

There are six key benefits to MASED: (i) a strong theoretical foundation in engineering physics, (ii) the ability to account for LV geometry and systolic pressure, (iii) correction for the effect of contractile stress on strain and vice versa, (iv) measurement of the physiologically significant parameter of work (in joules) per unit volume of myocardium), (v) integration of known LV risk factors, and (vi) early data suggesting higher prognostic discrimination compared with other LV metrics.

MASED provides a comprehensive assessment of myocardial mechanics, making it more advanced and informative than traditional indices. The two observational prognostic studies described indicate that GLASED could be important for risk stratification and guiding management decisions in cardiovascular medicine. Despite the added complexity, the addition of MASED into clinical studies may be warranted due to its associations with heart failure, all-cause mortality, and MACEs. We acknowledge the limitations of the retrospective analyses described and recommend large international prospective comparative survival studies by independent groups to confirm its clinical utility before widespread adoption can be recommended.

## Supplementary Material

qyaf105_Supplementary_Data

## Data Availability

Other information is available from DHM upon reasonable request.

## Lead author biography

Professor David MacIver is an accomplished clinical cardiologist with over 30 years of experience, serving as Director of Echocardiography at Musgrove Park Hospital in Taunton, UK. His clinical expertize spans coronary CT, heart failure and adult congenital heart disease. Since 2014, he has held a visiting professorship with the Biological Physics Group at the University of Manchester. Professor MacIver’s research focuses on the mechanisms of heart failure and the assessment of LV function, integrating mathematical and computational modelling with principles of engineering physics. Over the past two decades, he has led and contributed to numerous translational and clinical studies and has published extensively in peer-reviewed journals.
