# Supplementary material for: Myocardial active strain energy density and contractance: novel prognostic tools for left ventricular function and cardiovascular risk
Source: Eur Heart J Imaging Methods Pract. 2025 Oct 6;3(4):qyaf105. doi: 10.1093/ehjimp/qyaf105 (PMC12499770; doi:10.1093/ehjimp/qyaf105)
Supplement: qyaf105_Supplementary_Data [file qyaf105_supplementary_data.docx]

**Supplementary material**

**Table S1.** The table summarizes some of the key prognostic studies with populations, cutoffs, outcomes, hazard ratios, and study designs, which assessed potential structural or functional left ventricular prognostic metrics. Note most studies only compare one or two metrics.

| Metric | Study Population | Cutoff/Unit | Outcome | Adjusted HR (95% CI) | Study Design | Reference |
| --- | --- | --- | --- | --- | --- | --- |
| LVEF | Stable HF (n=7,788) | ≤15% vs 36-45% | All-cause mortality | 1.77 (1.48–2.11) | Prospective cohort | Curtis^^[[1]](#endnote-1)^^ |
|  | Australian observational registry | LVEF<40% (n=237,046) | All-cause mortality | Male 2.5; Female 2.8. | Retrospective observational | Stewart^[[2]](#endnote-2)^ |
| GLS | CRT candidates (n=82) | >–9% | Long-term mortality | 0.86 (0.75–0.99) | Multicentre prospective | Delgado^^[[3]](#endnote-3)^^ |
|  | Chronic HF (n=173) | Per SD change (–6.3%) | HF hospital/CV death | 2.15 (1.34–3.46) | Prospective cohort | Kamar^^[[4]](#endnote-4)^^ |
| GCS | DCM (n=70) | ≥5.4% | LV reverse remodelling | OR 7.69 (2.29–25.82) | Retrospective cohort | Tanaka^^[[5]](#endnote-5)^^ |
| LV Stroke Work Index | ITU (n=4,536) | <20 g.min/m^2^ | LVSWI better than LVEF | 2.4 with | Retrospective cohort | Jentzer^[[6]](#endnote-6)^ |
| Global Work Index (GWI) | DCM (n=116) | <788 mmHg% | MACE | 5.46 (1.66–17.92) | Retrospective cohort | Chen^^[[7]](#endnote-7)^^ |
|  | Advanced HF (n=105) | Per 100 mmHg% increase | Death/LVAD/Transplant | 0.85 (0.77–0.94) | Prospective observational | Hedwig^^[[8]](#endnote-8)^^ |
| LV Mass/BSA | Stable CHD (n=1,016) | >115 g/m² (M), >95 g/m² (F) | All-cause mortality | 2.0 (1.1–3.7) | Prospective cohort | Turaqhia^^[[9]](#endnote-9)^^ |
|  | Hypertensive (n=2,453) | High-stable trajectory | All-cause mortality | 3.0 (2.1–4.3) | Longitudinal observational | Zhou^^[[10]](#endnote-10)^^ |
|  | Cardiac patients (n=2,543) | Per 10 g/m² increase | Death/HF hospitalization | 1.16 (1.12–1.20) | Retrospective CMR | Lundin^^[[11]](#endnote-11)^^ |
| End-Systolic Volume Index | Post-CABG (n=193) | Per 20 mL/m² increase | 10-year mortality | 1.4 (1.1–1.8) | Longitudinal observational | Hamar^^[[12]](#endnote-12)^^ |
|  | Stable CHD (n=989) | >25 mL/m² | HF hospitalization | 4.6 (2.8–7.5) | Prospective cohort | McManus^[[13]](#endnote-13)^ |
| LV Global Function Index | NSTEMI (n=432) | <23.22 | 3-year mortality | 11.86 (3.60–39.10) | Retrospective cohort | Karatus^^[[14]](#endnote-14)^^ |
|  | ACS (n=1,499) | Lower tertile | 3-year MACE | Independent predictor | Retrospective cohort | Doganay^^[[15]](#endnote-15)^^ |
|  | Community (CARDIA, n>3,000) | Lower LVGFI | Incident HF, CVD | Independent predictor | Prospective cohort | Nwabuo^^[[16]](#endnote-16)^^ |
| Myocardial Contraction Fraction (MCF) | HCM (n=137) | Per 10% increase | Death/  transplant/CVE | 0.50 (0.28–0.90) | Prospective cohort | Shimada^^[[17]](#endnote-17)^^ |
|  | TAVR (n=400) | ≤30% | All-cause mortality | Independent predictor | Retrospective cohort | Romeo^^[[18]](#endnote-18)^^ |
|  | HFrEF (n=30) | Lower MCF | Functional capacity | Superior to LVEF | Prospective cohort | Abdellatif^^[[19]](#endnote-19)^^ |

Abbreviations:

**MACE**: Major Adverse Cardiovascular Events; **HR**: Hazard Ratio; **HCM**: Hypertrophic Cardiomyopathy; **HFrEF**: Heart Failure with Reduced Ejection Fraction.

**GLASED equation**

${GLASED=½ \times\sigma}_{z}\times\varepsilon_{z}$ (1)

*where* $\sigma_{z}$ is the longitudinal engineering stress, $\varepsilon_{z}$ is the peak longitudinal strain and

$\sigma_{z}=\frac{P{r_{i}}^{2}}{\left( {r_{o}}^{2}-{r_{i}}^{2} \right)}$ (2)

where *P* is the brachial systolic blood pressure*,* $r_{i}$ is the internal end-diastolic radius, and $r_{o}$ is the outer end-diastolic radius.

**CASED equation**

*CASED* =${½ \times\sigma_{\theta}}\times{}_{\theta}$ (3)

where $\sigma_{\theta}$ is the circumferential stress and where $\varepsilon_{\theta}$ is the circumferential strain and

$\sigma_{\theta} = \frac{P{r_{i}}^{2}}{\left( {r_{o}}^{2}-{r_{i}}^{2} \right)} \times\frac{{r_{i}}^{2}{r_{o}}^{2}\left( P \right)}{{r_{m}}^{2}\left( {r_{o}}^{2}-{r_{i}}^{2} \right)}$ (4)

**SASED equation**

$SASED=GLASED+CASED$ (5)

**Comparison of myocardial work index (MWI) and strain energy density**

*S*$ED\left( u \right)=\int\sigma d\varepsilon$ $\sigma=f(P,G)$ (6)

$$where \sigma is a function of both pressure\left( P \right) and geometry\left( G \right)$$

$SE (U)= P\times G\times\varepsilon\times V$ (7)

*Myocardial work index =*$\int Pd\varepsilon$

$MWI=\int Pd\varepsilon$*≠*$\int\sigma d\varepsilon\times V$ *(only works if* $G\times V=1$) (8)

Where MWI index is a function of luminal pressure not pressure and geometry. The geometric factor part of the stress equation (in brown in Manuscript Figure 2) is absent and so the MWI will only be indexed to work when the LVIDD and EDWT give the same geometric factor part of the stress equation.

**Derivation of pressure-volume loops from work**

Work is the energy transferred to an object via the application of force through displacement. The mechanical work/energy (*W*), measured in Joules (J), is defined as

$W=F\cdot\boldsymbol{\Delta s}=\boldsymbol{F\Delta s}Cos \theta$ (9)

where ***F*** is the length of a constant vector force, $\boldsymbol{\Delta}$***s*** is the distance the object is displaced, and $\theta$ is the angle between the force and displacement vectors. For the dot product of F and $\boldsymbol{\Delta s}$ then$\mathrm{Cos}\theta$ = 1 when the directions of *F* and $\Delta$*s* are the same and can be treated as scalar quantities.

Therefore,

$F=P\times A$ (10)

where *P* is the pressure and *A* is the area on which the pressure is exerted.

The change in volume ($\Delta V)$ is the area (*A*) multiplied by the displacement $\Delta$*s,* i.e.,

$\Delta V=A\times\Delta s$ (11)

Therefore,

$W=F\times\Delta s$ (12)

$=P\times A\times\Delta s$ (13)

$=P\times\Delta V$ (14)

where *P* changes with respect to *V;* then, it is written as

$$W=\oint_{V_{1}}^{V_{2}} P_{V}dV$$

**Derivation of strain energy density (u) from work**

$u=\frac{W}{V}=\frac{W}{L_{0}\times A}$ (15)

substituting Equation (4) and where *V* is the total material volume (${V=L}_{0}\times A$)

$=\frac{F\times\Delta s}{L_{0}\times A}$ (16)

where the length is $L_{0}$ is the original length and $V=L_{0}\times A$, rearranged

$=\frac{F}{A}\times\frac{\Delta s}{L_{0}}$ (17)

Stress $(\sigma)=\frac{F}{A}$ and strain $(\varepsilon)=\frac{\Delta s}{L_{0}}$, substituting into (8)

Therefore, the strain energy density (*u*) for a *constant* stress is

$u=\sigma\times\varepsilon$ (18)

The strain energy (U), mechanical work done or stored in the deformed material is

$$U=u\times V=\sigma\times\varepsilon\times V$$

where the stress changes with strain, and then the strain energy density is written as follows:

$u=\int_{\epsilon_{0}}^{\epsilon_{1}} \sigma_{\epsilon}d\epsilon$ (19)

where $\epsilon_{0}$ is the starting strain (usually 0) and where $\epsilon_{1}$ is the final or peak strain.

**Figure S1. Comparison of area under the curve using numerical integration and analytic method using equation in ex vivo preparations.**

*
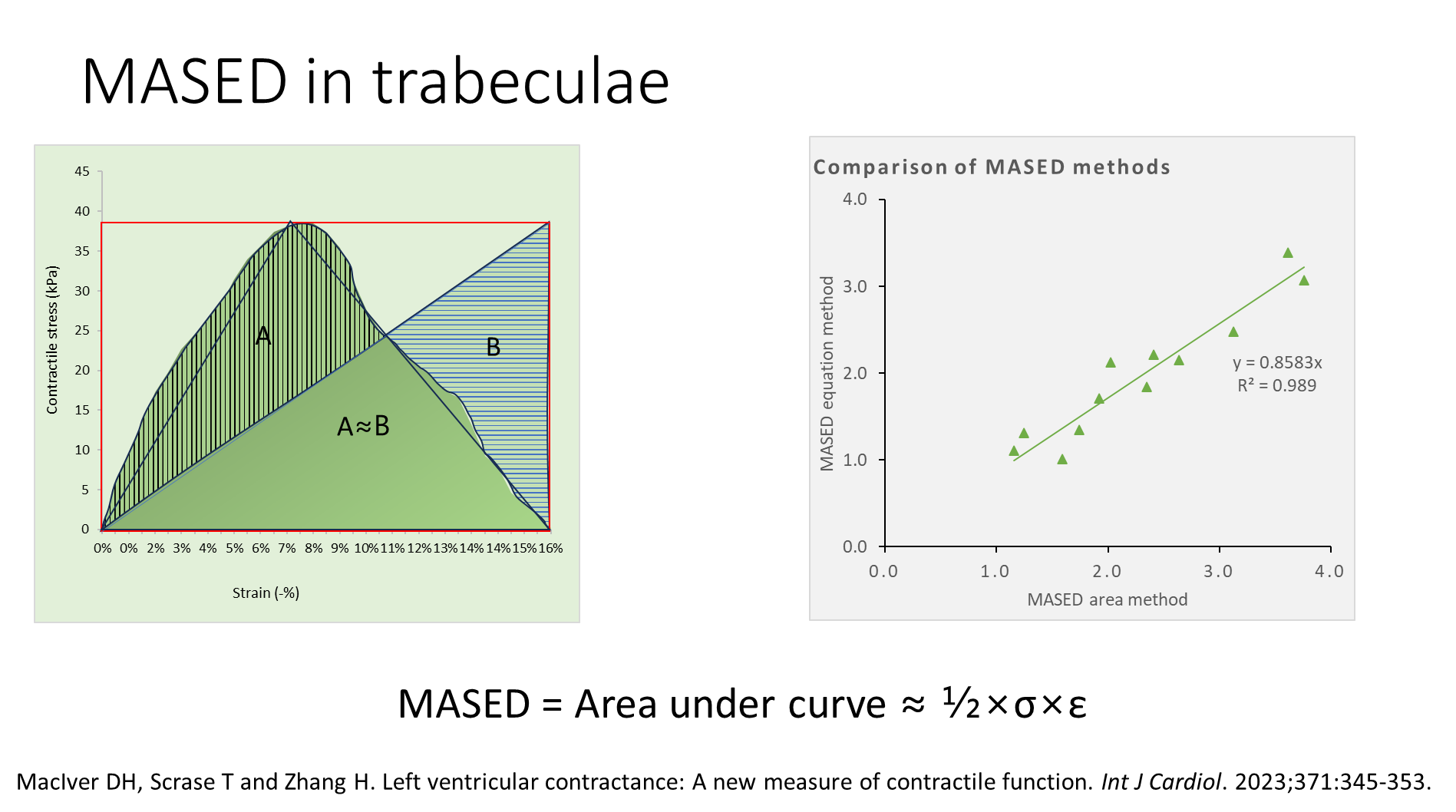
*

Left hand panel shows the stress-strain relationship used to calculate contractance (MASED) in ex vivo trabeculae. The area within the stress-strain loop (green area) provides the contractance measured in kJ/m^3^. The triangular area B is ½ the area of the rectangle shown in red (i.e., peak stress times peak strain). The dark green area is similar to triangle A and the area of triangle A is the same as the area of triangle B. The right panel shows the correlation between the green area (numerical integration) and the equation (analytic) methods (area B).^10^

**Figure S2. Comparison of area under the curve using numerical integration and analytic method using equation in the in vivo study.**

*
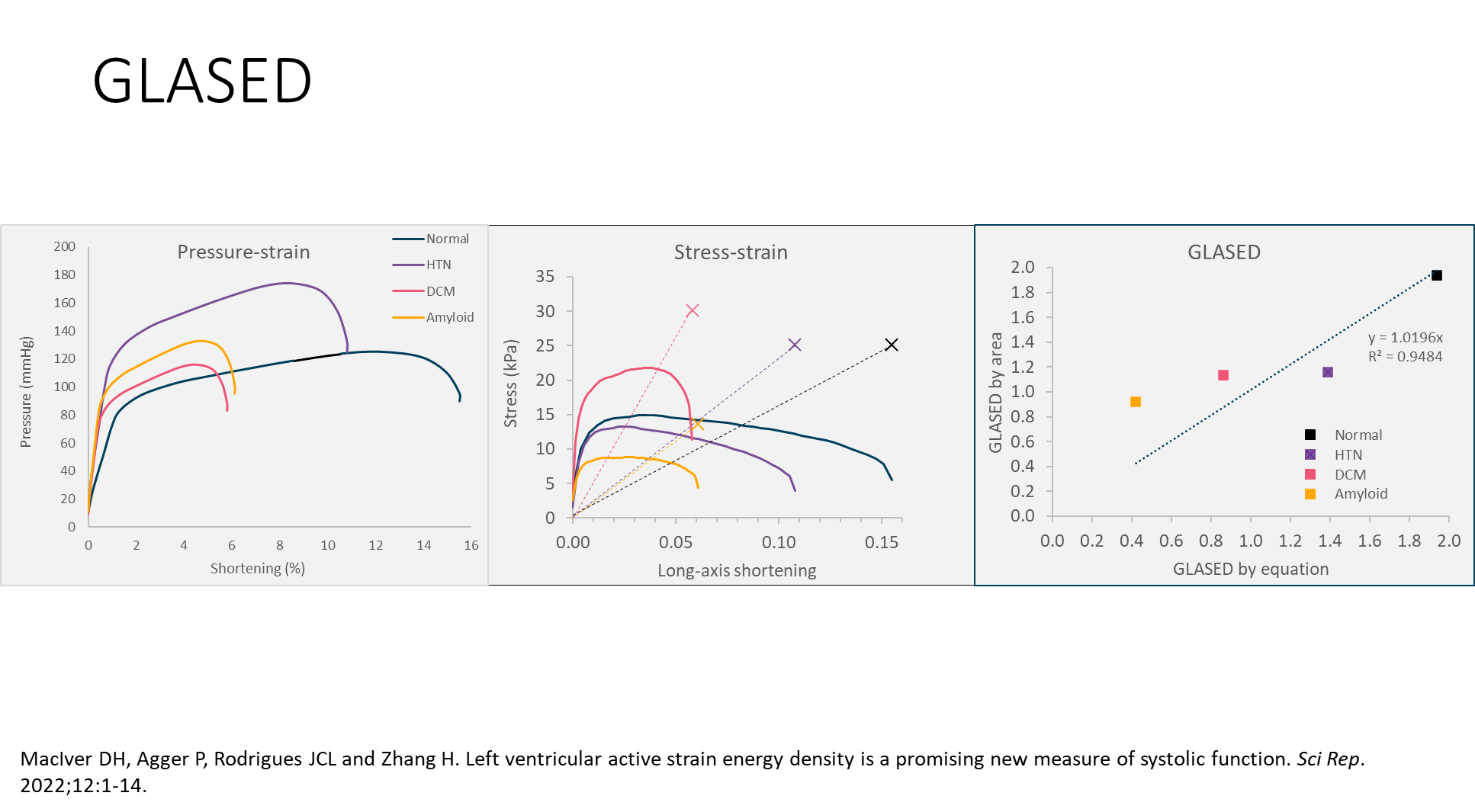
*

The panels below show the calculation of GLASED comparing the area under the curve method (numerical integration) with the equation (analytic) method. The left panel shows the pressure-strain curves, the middle panel the stress-strain curves. Note the marked difference in pressure-strain and stress strain curves. The right panel compares the area method with the equation method and shows a good correlation between the two when engineering stress (i.e. based on systolic pressure) is used in the equation (middle panel - crosses).^12^

**Figure S3A. Effect of changes in stress-strain relationship with altered afterload in ex vivo trabecular tissue**


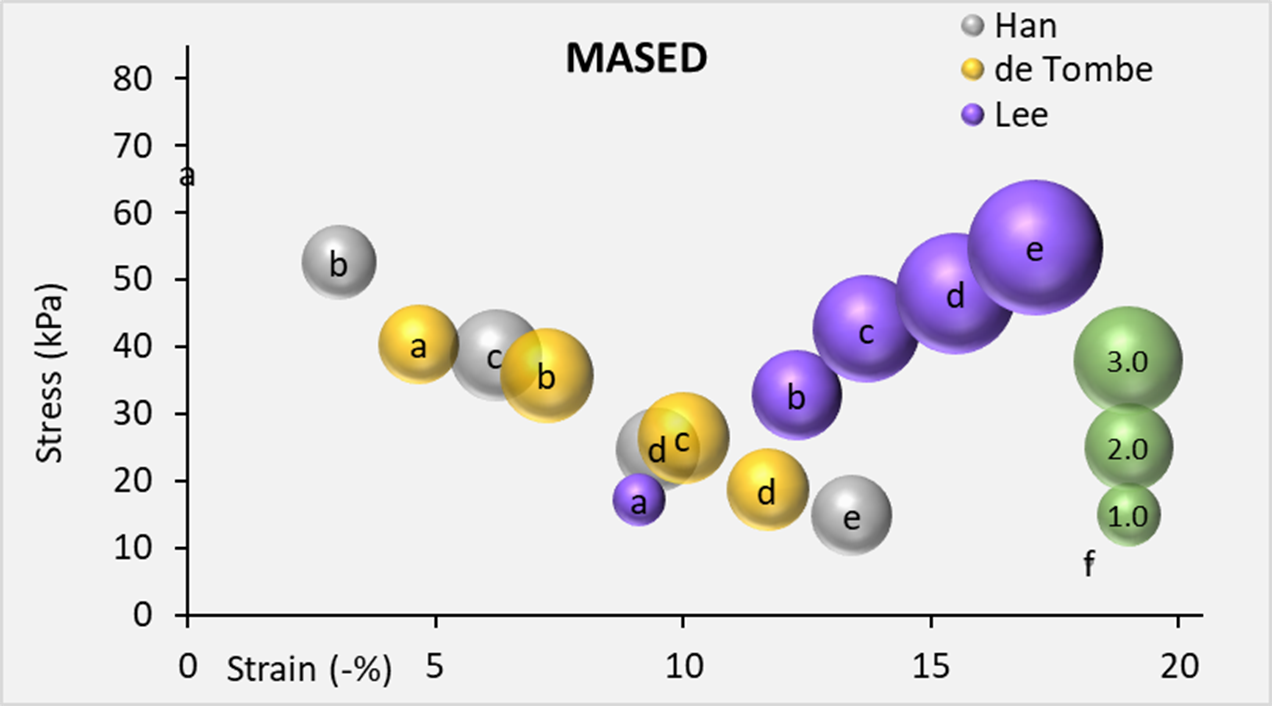


Note that there is a negative linear relationship between stress and strain as afterload is altered in two experiments (grey and gold spheres). With increasing inotropy, stress, strain and contractance gradually increase in a linear manner (purple spheres).^10^ The size spheres indicate the magnitude of MASED (contractance) in kJ/m^3^ (green spheres).

**Figure S3B. Charts showing the optimal working stresses and strains for ex vivo trabecular tissue.**


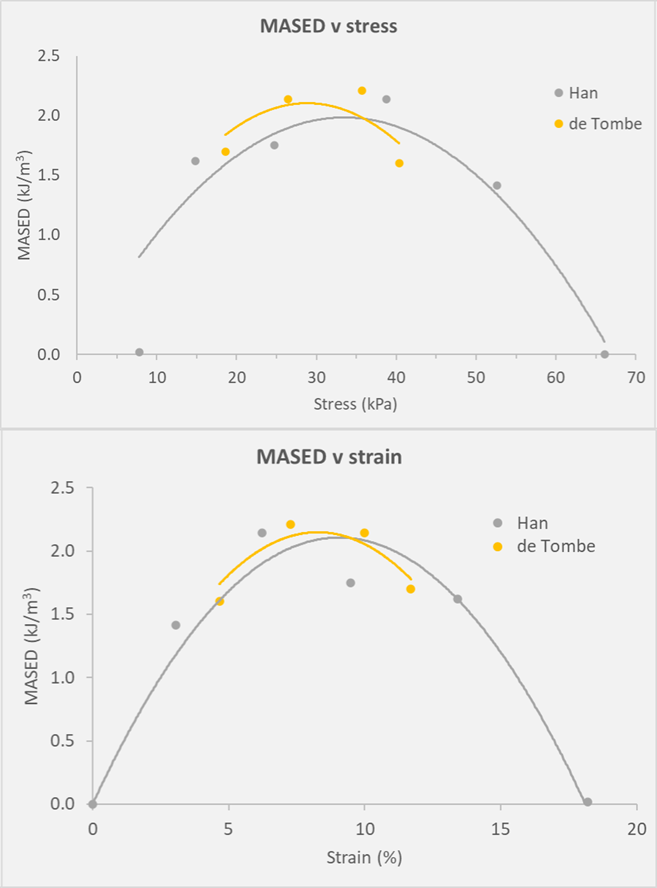


Note the optimal contractance for ex vivo trabecular tissue is at a strain of about -8% and contractile stress at about 30 kPa.^10^ The higher stress demands results in a lower strain above the optimal contractile stress ability or capacity (a stress demand-capacity mismatch).

**Figure S4: Effect of input variables on MASED and integration of risk factors**

**
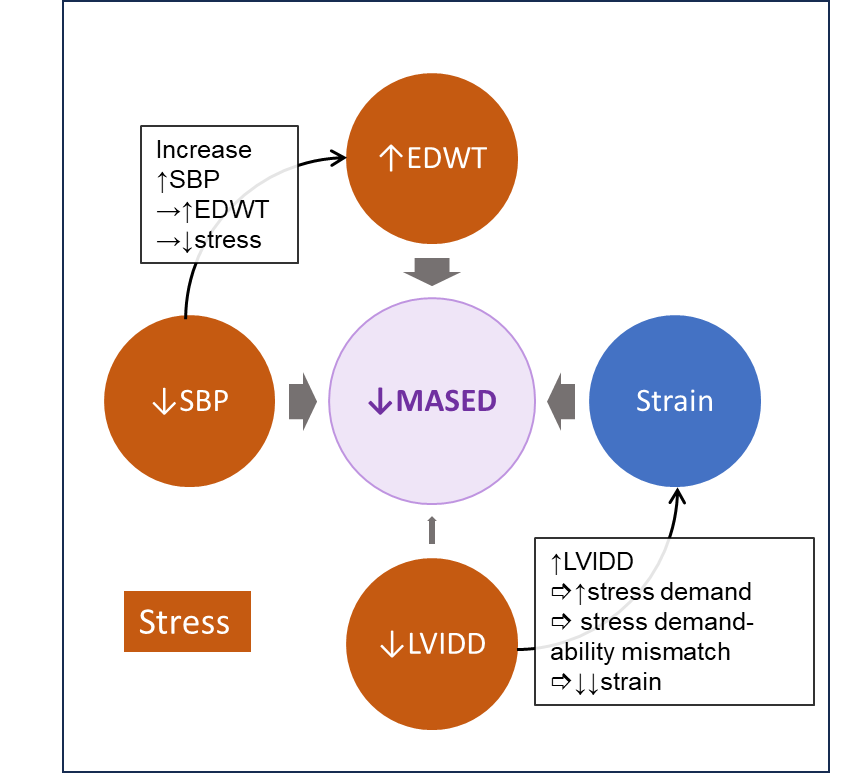
**

Figure shows the direct and indirect mechanisms that reduce MASED. A lower SBP, LVIDD and strain each independently decrease MASED. A greater EDWT also decreases MASED. Hypertension increases MASED but an accompanying increase in EDWT decreases MASED. An isolated increase in LVIDD increases stress demand without increasing contractile stress ability or capacity (despite additional adrenergic drive) leading to a demand-ability mismatch and resulting in a marked fall in strain and therefore a significant fall in MASED. Width of the arrows represent the relative impact of the input variables (risk factors) on MASED.

**Figure S5. Mathematical modelling of the effect of the input variable on GLASED.**


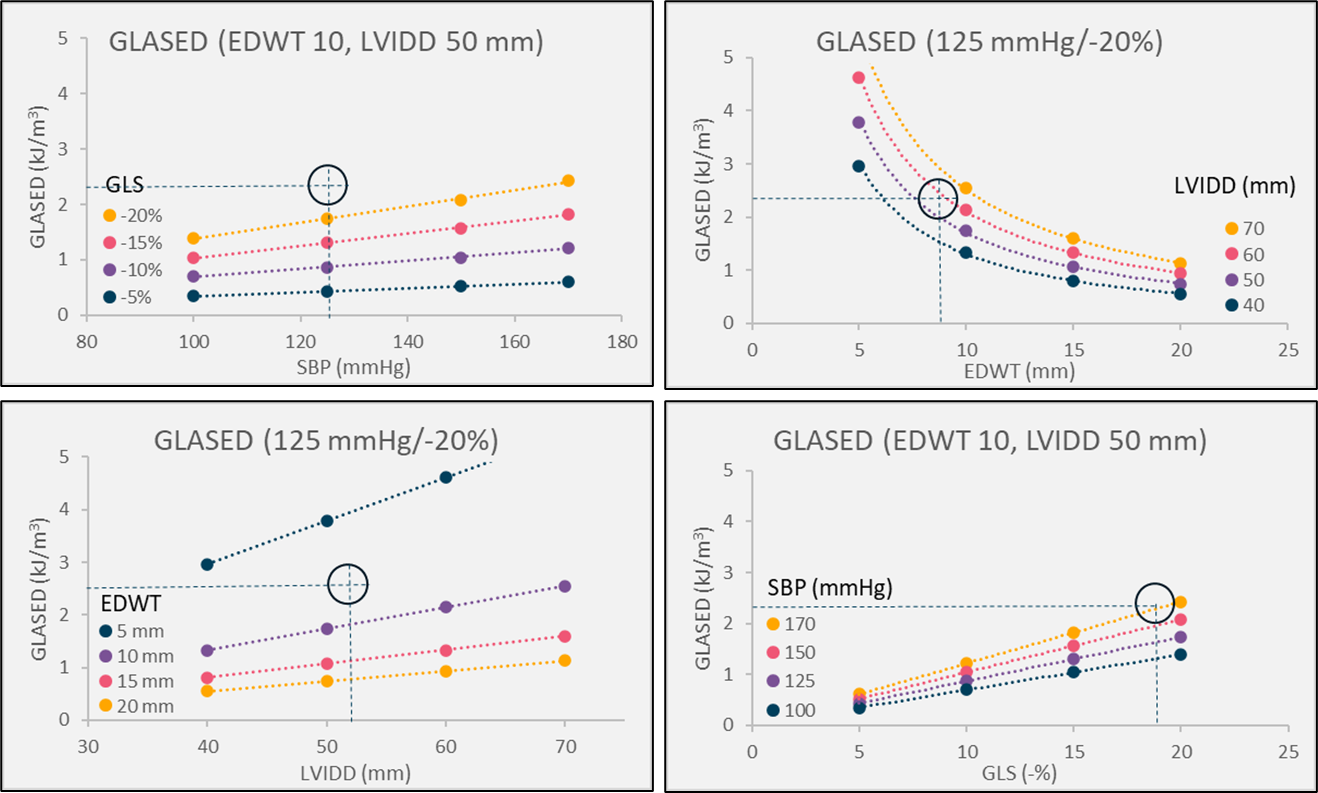


The top left graph shows the family of linear relationship of systolic blood pressure and GLASED with varying GLS. The top right graph shows the influence of LV wall thickness on GLASED with varying LVIDD; note how GLASED decreases rapidly in a curvilinear pattern which is most marked between 5 mm and 10 mm thickness. Bottom left graph shows the linear increasing effect of LVIDD on GLASED with varying EDWT. Bottom right graph shows the linear effect of GLS on GLASED with varying SBP. Circles represent the normal GLASED.

**Estimates of propagation errors**

Error propagation was assessed using Monte Carlo simulation (100,000 iterations) and sensitivity weighting. Increasing measurements from a single measurement of each variable to 3 SBP, 12 end-diastolic wall thickness measurements, 6 LVIDD measurements, and 3 GLS measurements reduced the coefficient of variation from 20.9% to 8.8% (Table S3).

**Table S2 Coefficient of variation of single vs. multiple measurements on input variables and GLASED**

| Variable | Single | Multiple |
| --- | --- | --- |
| SBP | 5.0% | 5.00% |
| LVIDD | 8.0% | 3.27% |
| EDWT | 10.0% | 2.89% |
| GLS | 8.0% | 3.27% |
| GLASED | 20.9% | 8.8% |

Assumes Error_multiple = Error_single / √n.

**Table S3. GLASED reference ranges (95%) for echocardiography (MacIver et al. 2024)**

| Population | N | Mean ± SD (kJ/m³) | Lower Limit | Upper Limit |
| --- | --- | --- | --- | --- |
| All participants | 447 | 2.27 ± 0.48 | 1.34 | 3.21 |
| Young male athletes | 245 | 2.40 ± 0.42 | 1.58 | 3.22 |
| Young female athletes | 67 | 2.28 ± 0.41 | 1.48 | 3.09 |
| Veteran male athletes | 70 | 1.96 ± 0.51 | 1.02 | 2.91 |
| Veteran female athletes | 44 | 1.92 ± 0.51 | 0.92 | 2.93 |
| Male controls | 21 | 2.31 ± 0.39 | 1.54 | 3.08 |

**Table S4. GLASED reference ranges (95%) for CMR in a community-based cohort (Aung et al. 2024)**

| Population | N | Mean ± SD (kJ/m³) | Lower Limit | Upper Limit |
| --- | --- | --- | --- | --- |
| All participants | 44,957 | 2.60 ± 0.60 | 1.42 | 3.78 |
| CVD-free | 41,091 | 2.57 ± 0.55 | 1.49 | 3.65 |
| With CVD | 3,866 | 2.42 ± 0.58 | 1.28 | 3.56 |

**Table S5. GLASED reference ranges (95%) for CMR in tertiary refereal centre (MacIver et al. 2022).**

| Population | N | Mean ± SD (kJ/m³) | Lower Limit | Upper Limit |
| --- | --- | --- | --- | --- |
| Normal controls  (45±14 years) | 39 | 2.27 ± 0.57 | 1.16 | 3.38 |

Note these are rescaled from original data which used longitudinal shortening to an estimate of GLS by mulitiplying the former by 1.17 to achieve a mean GLS of -18.1% .

**Supplementary references**

1. Curtis, J, Sokol, S, Wang, Y. et al. The association of left ventricular ejection fraction, mortality, and cause of death in stable outpatients with heart failure. JACC. 2003 Aug, 42 (4) 736–742. [↑](#endnote-ref-1)
2. Stewart S, Playford D, Scalia GM, Currie P, Celermajer DS, Prior D, Codde J, Strange G; NEDA Investigators. Ejection fraction and mortality: a nationwide register-based cohort study of 499 153 women and men. Eur J Heart Fail. 2021 Mar;23(3):406-416. [↑](#endnote-ref-2)
3. A. Delgado-Montero, B. Tayal, A. Goda, K. Ryo, J. J. Marek, M. Sugahara, et al. Additive Prognostic Value of Echocardiographic Global Longitudinal and Global Circumferential Strain to Electrocardiographic Criteria in Patients With Heart Failure Undergoing Cardiac Resynchronization Therapy Circulation: Cardiovascular Imaging 2016 Vol. 9 Issue 6 Pages e004241 [↑](#endnote-ref-3)
4. Abou Kamar S, Aga YS, de Bakker M, van den Berg VJ, Strachinaru M, Bowen D, Frowijn R, Akkerhuis KM, Brugts J, Manintveld O, Umans V, Geleijnse ML, Boersma E, van Dalen BM, Kardys I. Prognostic value of temporal patterns of global longitudinal strain in patients with chronic heart failure. Front Cardiovasc Med. 2023 Jan 12;9:1087596. [↑](#endnote-ref-4)
5. Tanaka H, Matsumoto K, Sawa T, Miyoshi T, Motoji Y, Imanishi J, Mochizuki Y, Tatsumi K, Hirata K. Evaluation of global circumferential strain as prognostic marker after administration of β-blockers for dilated cardiomyopathy. Int J Cardiovasc Imaging. 2014 Oct;30(7):1279-87 [↑](#endnote-ref-5)
6. Jentzer JC, Anavekar NS, Burstein BJ, Borlaug BA, Oh JK. Noninvasive Echocardiographic Left Ventricular Stroke Work Index Predicts Mortality in Cardiac Intensive Care Unit Patients. Circ Cardiovasc Imaging. 2020 Nov;13(11):e011642. [↑](#endnote-ref-6)
7. Chen, P., Aurich, M., Greiner, S., Maliandi, G., Müller-Hennessen, M., Giannitsis, E., Meder, B., Frey, N., Pleger, S., Mereles, D., 2024. Prognostic relevance of global work index and global constructive work in patients with non-ischemic dilated cardiomyopathy. The International Journal of Cardiovascular Imaging 40, 1575–1584. [↑](#endnote-ref-7)
8. F. Hedwig, O. Nemchyna, J. Stein, C. Knosalla, N. Merke, F. Knebel, et al. Myocardial Work Assessment for the Prediction of Prognosis in Advanced Heart Failure. Front Cardiovasc Med 2021 Vol. 8 Pages 691611 [↑](#endnote-ref-8)
9. Turakhia MP, Schiller NB, Whooley MA. Prognostic significance of increased left ventricular mass index to mortality and sudden death in patients with stable coronary heart disease (from the Heart and Soul Study). Am J Cardiol. 2008 Nov 1;102(9):1131-5. [↑](#endnote-ref-9)
10. Zhou D, Huang Y, Cai A, Yan M, Cheng Q, Feng X, Nie Z, Feng Y. Longitudinal Study of Left Ventricular Mass Index Trajectories and Risk of Mortality in Hypertension: A Cohort Study. J Am Heart Assoc. 2023 May 2;12(9):e028568. [↑](#endnote-ref-10)
11. Lundin, M., Heiberg, E., Nordlund, D. et al. Prognostic utility and characterization of left ventricular hypertrophy using global thickness. Sci Rep 13, 22806 (2023 [↑](#endnote-ref-11)
12. Hamer AW, Takayama M, Abraham KA, Roche AH, Kerr AR, Williams BF, Ramage MC, White HD. End-systolic volume and long-term survival after coronary artery bypass graft surgery in patients with impaired left ventricular function. Circulation. 1994 Dec;90(6):2899-904. [↑](#endnote-ref-12)
13. McManus DD, Shah SJ, Fabi MR, Rosen A, Whooley MA, Schiller NB. Prognostic value of left ventricular end-systolic volume index as a predictor of heart failure hospitalization in stable coronary artery disease: data from the Heart and Soul Study. J Am Soc Echocardiogr. 2009 Feb;22(2):190-7. [↑](#endnote-ref-13)
14. Karatas M, Sabanoglu C, Sahin KE, Inanc IH. Left Ventricular Global Function Index: A Potential Predictor of Mortality and Major Adverse Cardiovascular Events in NSTEMI Patients. Medicina (Kaunas). 2025 Mar 11;61(3):487. [↑](#endnote-ref-14)
15. Doganay B, Celebi OO. Prognostic role of the left ventricular global function index in predicting major adverse cardiovascular events in acute coronary syndrome patients. Biomark Med. 2023 Jan;17(1):5-16. [↑](#endnote-ref-15)
16. Nwabuo CC, Moreira HT, Vasconcellos HD, Mewton N, Opdahl A, Ogunyankin KO, Ambale-Venkatesh B, Schreiner PJ, Armstrong AAC, Lewis CE, Jacobs DR, Lloyd-Jones D, Gidding SS, Lima JAC. Left ventricular global function index predicts incident heart failure and cardiovascular disease in young adults: the coronary artery risk development in young adults (CARDIA) study. Eur Heart J Cardiovasc Imaging. 2018;20(5):533-540. [↑](#endnote-ref-16)
17. Shimada YJ, Hoeger CW, Latif F, Takayama H, Ginns J, Maurer MS. Myocardial Contraction Fraction Predicts Cardiovascular Events in Patients With Hypertrophic Cardiomyopathy and Normal Ejection Fraction. J Card Fail. 2019 Jun;25(6):450-456. [↑](#endnote-ref-17)
18. Romeo, F.J., Seropian, I.M., Arora, S., Vavalle, J.P., Falconi, M., Oberti, P., Kotowicz, V., Agatiello, C.R., Berrocal, D.H. Prognostic impact of myocardial contraction fraction in patients undergoing transcatheter aortic valve replacement for aortic stenosis. Cardiovascular Diagnosis and Therapy 2020, 10, 12–23. [↑](#endnote-ref-18)
19. Y. A. Abdellatif, H. A. Addow and R. R. Elias. Myocardial Contraction Fraction is Superior to Ejection Fraction in Predicting Functional Capacity in Patients with Heart Failure with Reduced Ejection Fraction J Saudi Heart Assoc 2022 Vol. 34 (1)15-23. [↑](#endnote-ref-19)
